# A Push to Consider Mantle Cell Lymphoma in Adults with Leukemia/Lymphoma with Blastoid Morphology

**DOI:** 10.3390/hematolrep15040061

**Published:** 2023-10-13

**Authors:** Nkechi Arinze, Nivin Omar, Amany Keruakous, Ravindra Kolhe, Natasha Savage

**Affiliations:** Medical College of Georgia, Augusta University, Augusta, GA 30912, USA

**Keywords:** mantle cell lymphoma, diffuse large B-cell lymphoma, FISH, SOX11, flow cytometry, Non-Hodgkin’s Lymphoma

## Abstract

Mantle cell lymphoma (MCL) is an intermediate-grade B-cell lymphoma, representing 2.8% of all non-Hodgkin lymphomas in the US. It is associated with t(11;14)(q13; q23), which leads to the overexpression of cyclin D1, consequently promoting cell proliferation. MCL usually expresses CD19, CD20, CD43, surface immunoglobulins, FMC7, BCL2, cyclin D1, CD5, and SOX11. Herein is a case of a 67-year-old male, referred to our facility with shortness of breath, anemia (hemoglobin of 5.3 g/dL), thrombocytopenia (12 × 10^9^/L), and leukocytosis (283 × 10^9^/L). A peripheral blood smear showed marked lymphocytosis with blastoid morphology. Morphologic examination of the bone marrow biopsy revealed a diffuse sheet of blastoid cells expressing CD20 and CD10, but without CD5 or cyclin D1. Given these features, a differential diagnosis of diffuse large B-cell lymphoma (DLBCL) with germinal center derivation, high-grade follicular lymphoma, and Burkitt lymphoma was considered, with the latter not favored due to morphology. Additional studies revealed positive SOX11, and fluorescence in situ hybridization (FISH) studies detected t(11;14). These additional studies supported diagnosis of the blastoid variant of MCL. In conclusion, we present a unique and challenging case of MCL without cyclin D1 or CD5, but with an expression of CD10 and SOX11, along with t(11;14). Pathologists should explicitly consider the blastoid variant of MCL when dealing with mature B-cell neoplasms with blastoid morphology in adults, and utilize a broad panel of ancillary studies, including FISH and SOX11.

## 1. Introduction

Mantle cell lymphoma (MCL) is an intermediate-grade B-cell lymphoma [1]. It constitutes approximately 3–10% of adult-onset non-Hodgkin lymphoma (NHL) in Western countries [2]. Moreover, an incidence of approximately four to eight cases per million persons per year is reported in the United States [2]. In Asian countries, the incidence of MCL is variable (1–6% of all lymphomas) [3]. There is a reported increasing incidence with age; the median age at diagnosis is 68 years. A male predilection is noted [2].

Lymph nodes are the most involved site. The characteristics of classical MCL usually involves excessive growth of monotonous lymphoid cells leading to expansion of the mantle zone around the germinal center, with frequent subsequent effacement of the normal nodal architecture [3]. In addition, aggressive morphological variants that pose a diagnostic dilemma have been described, including the blastoid and pleomorphic variants [3]. The blastoid variant of MCL have large lymphoma cells, with dispersed chromatin, a high proliferative index, (Ki-67), and high mitotic activity (≥20–30 mitoses per 10 high-power fields), whereas the lymphoma cells of the pleomorphic variant of MCL show nuclear pleomorphism, large, to oval nuclei with irregular nuclear contours, generally pale cytoplasm, and often with prominent nucleoli and pale cytoplasm [3].

The majority of MCL is associated with t(11;14)(q13; q23) between immunoglobulin heavy chain (IGH) and CCND1, which leads to the overexpression of cyclin D1, consequently promoting cell proliferation. MCL usually expresses CD19, CD20, CD43, surface immunoglobulins, FMC7, BCL2, cyclin D1, CD5, and SOX11 [3].

We present a unique and challenging case of MCL without cyclin D1 or CD5, but with an expression of CD10 and SOX11, as well as t(11;14). Pathologists should consider the blastoid variant of MCL when dealing with mature B-cell neoplasms with blastoid morphology in adults, and therefore utilize a broad panel of ancillary studies, including FISH and SOX11 immunohistochemical staining.

## 2. Clinical Case Presentation

**Figure 1:**
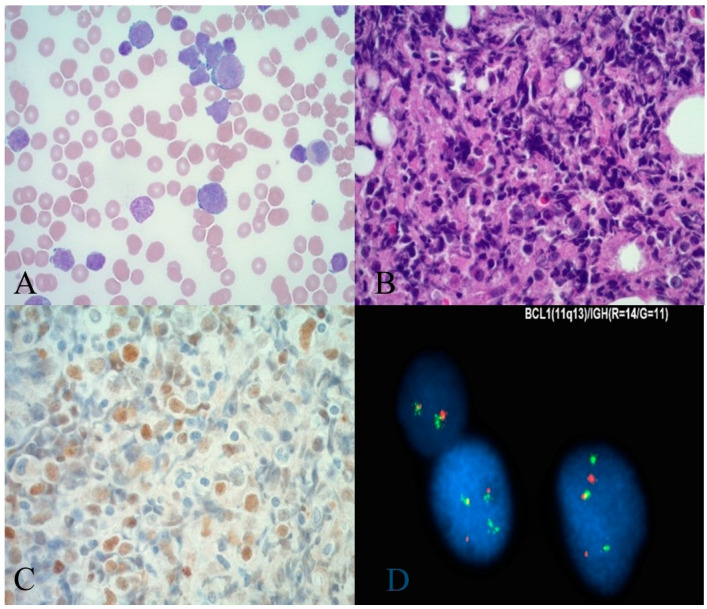
(**A**)—Peripheral blood smear showing marked lymphocytosis and blastoid morphology, (**B**)—Bone marrow biopsy with sheets of blastoid cell in a diffuse pattern hematoxylin and eosin x40, (**C**)—Immunohistochemistry showing SOX 11 positivity; (**D**)—FISH studies demonstrating t(11;14).

The patient is a 67-year-old male who was referred to our facility with complaints of dyspnea, fatigue, anemia (5.3 g/dL), thrombocytopenia (12 × 10^9^/L), and leukocytosis (283 × 10^9^/L). The patient also had an elevated serum lactate dehydrogenase level (LDH) of 1102 U/L. A peripheral blood smear showed marked lymphocytosis with blastoid morphology (Figure 1A).

**Figure 2:**
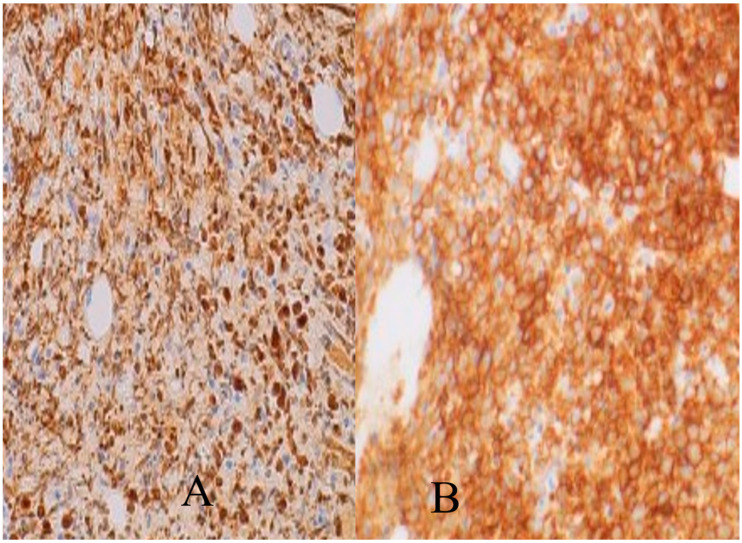
(**A**) Lymphoma cells with greater than 60% Ki67, (**B**) Immunohistochemistry showing CD10 positive cells.

Imaging of the chest, abdomen, and pelvis showed generalized lymphadenopathy and splenomegaly. Magnetic resonance imaging (MRI) of the brain was remarkable for dural smoothening, prompting concern for leptomeningeal disease. Morphologic examination of the bone marrow biopsy revealed a sheet-like growth of blastoid cells in a diffuse pattern. Flow cytometric immunophenotyping revealed a light chain-restricted, monotypic B-cell population expressing CD10 (Figure 2B) without CD5. Immunohistochemical analysis of this infiltrate initially revealed a CD10-positive B-cell lymphoma without cyclin D1; subsequent staining was positive for SOX11 (Figure 1B–D). Ki-67 highlighted >60% of the lymphoma cells (Figure 2A). Per FISH studies, t(11;14) was detected in the vast majority of cells. The patient was commenced on appropriate chemotherapy including pre-phase cyclophosphamide, a hyper-CVAD regimen, and intrathecal chemotherapy. The patient was doing well initially, as suggested by the resolution of his fevers, and altered mental status, stable vital signs, and resolving leukocytosis (3 × 109/L) but then developed sepsis due to an extended beta-lactamase Klebsiella infection with subsequent multiorgan failure, resulting in his demise.

## 3. Discussion

The current World Health organization (WHO) classification of MCL describes three subtypes: in situ MCL, MCL, and leukemic non-nodal MCL [4]. It is helpful to make a diagnostic distinction between the blastoid (and pleomorphic) variant of MCL and the classic/typical form, as the blastoid variant of MCL is an aggressive disease that poses a diagnostic challenge and shows a poor response to most available chemotherapies [3]. The blastoid variant of MCL has a distinct morphology that differentiates it from classic MCL, namely blastoid MCL reveals blastic features with a high Ki-67 [3]. Blastoid MCL morphology is characterized by roundish nuclei with a narrow rim of cytoplasm and finely dispersed chromatin. However, the pleomorphic variant has relatively large, pleomorphic oval-to-round nuclei with prominent nucleoli appearing like that of diffuse large B-cell lymphoma, which poses a diagnostic dilemma [3,5].

The blastoid variant of MCL accounts for approximately 30% of MCLs, and mostly involves males in their sixth decade of life [3]. It exhibits extra nodal involvement with advanced disease at presentation, and frequently involves the central nervous system (CNS) [5,6], as observed in our case. As evidence, one study reported a higher rate of CNS involvement in the blastoid variant of MCL. CNS involvement observed in 28% of blastoid MCL compared to 10% in the classic MCL with a statistical significance of; *p* < 0.0001. Additional risk factors for CNS involvement includes B-symptoms and increased serum LDH [7].

The molecular characteristics of MCL is the t(11;14)(q13;q32) leading to the rearrangement of the CCND1 gene to the immunoglobulin heavy chain (IGH) gene, resulting in cyclin D1 overexpression [2], along with the expression of CD19, CD20, CD43, surface immunoglobulins, FMC7, BCL2, cyclin D1, CD5, and SOX11 [3]. MCL also shows aberrant phenotypes including the loss of CD5 expression in about 6%of the cases [8] and the expression of CD10 in about 3.8% of the cases [9]. Our case is rare and falls within the phenotype mentioned above, due to the loss of cyclin D1 and CD5 expression, and the aberrant expression of CD10. However, SOX11 was positive and t(11:14) was detected, confirming MCL.

The CCND1-negative blastoid variant of MCL is relatively rare [10] and poses diagnostic and therapeutic difficulty. Salaverria et al. reported that most of the CCND1-negative MCLs expressed SOX11 with CD5 positivity, and a loss of CD10. Only one patient in their study series had blastoid MCL at presentation, accounting for approximately 3%. SOX11 has been shown to be a reliable marker in MCL with and without CCND1-expression [10]. This further reflects the rarity of this disease entity. CCND2 rearrangements were observed in most of these patients, accounting for approximately 55% of the gene alterations in this rare subgroup. Given the immunohistochemical and morphologic features of CCND1-negative MCL, there is a chance that many CCND1-negative blastoid MCLs have been erroneously misclassified as CD5-negative DLBCL [10].

Two cases of the CD5-negative blastoid variant of MCL have been reported in a case series by Yamamoto N et al. These cases presented as CD5-negative DLBCL on relapse, necessitating further evaluation. A composite re-evaluation, including bone marrow morphology, immunohistochemistry, and FISH testing, revealed t(11:14). These patients were placed on appropriate chemotherapy treatments and achieved complete remission. This prompted a review of all patients diagnosed with CD5-negative DLBCL. In their study, CD5 negativity accounted for 15% of the blastoid variant of MCL [3].

The current treatment regimen for the blastoid variant of MCL includes cytarabine-based chemotherapy. The bendamustine chemotherapeutic agent used in treating classical variants has minimal therapeutic effect on the blastoid variants [5]. Other novel therapeutic regimens with ibrutinib, venetoclax, temsirolimus, and lenalidomide have also been described in the management of blastoid MCL [5,11,12]. Stem cell transplant therapy, both autologous and allogeneic, has also been described [5,13]. The blastoid variant of MCL has been shown to demonstrate a lower treatment response and poor overall survival [14]. This could be attributed to the complex genomic characteristics and aberrant cell signaling pathways different from classic MCL [14]. Our patient received an appropriate chemotherapy regimen, including prephase cyclophosphamide, a hyper-CVAD regimen, and intrathecal chemotherapy. He was initially doing well as suggested by the resolution of fevers and altered mental status, stable vital signs, and resolving leukocytosis (3 × 109/L) until he, unfortunately, developed sepsis, which was consequently complicated by multiorgan failure, resulting in his demise.

## 4. Conclusions

In conclusion, we present a unique and challenging case of the blastoid variant of MCL without cyclin D1 or CD5, but with an expression of CD10 and SOX11, along with t(11;14). Pathologists should be aware of the blastoid variant of MCL when dealing with mature B-cell neoplasms with blastoid morphology, and ought to include a broad panel of ancillary studies, including FISH for the detection of t(11;14) and immunohistochemistry to analyze SOX11. Specifically, in adults presenting with mature leukemia/lymphoma with blastoid morphology, blastoid MCL should be high on the differential diagnosis, and potentially preferred as the diagnosis until proven otherwise by a lack of cyclin D1, along with SOX11, t(11;14), and CCND2 rearrangements. An appropriate and prompt diagnosis of the blastoid variant carries remarkable therapeutic and prognostic significance. We also recommend more research and investigations on the pathogenesis, pathophysiology, and treatment of this disease to further understand and improve on the current treatment regimen.

## Data Availability

Not applicable.
